# Exploration of the binding determinants of protein phosphatase 5 (PP5) reveals a chaperone-independent activation mechanism

**DOI:** 10.1016/j.jbc.2024.107435

**Published:** 2024-06-01

**Authors:** Shweta Devi, Annemarie Charvat, Zoe Millbern, Nelson Vinueza, Jason E. Gestwicki

**Affiliations:** 1 Department of Pharmaceutical Chemistry and the Institute for Neurodegenerative Diseases, University of California San Francisco, San Francisco, California, USA; 2 Department of Textile Engineering, North Carolina State University, Raleigh, North Carolina, USA

**Keywords:** short linear motifs, proteostasis, protein–protein interactions, positional scanning combinatorial library, C-end rule

## Abstract

The protein phosphatase 5 (PP5) is normally recruited to its substrates by the molecular chaperones, heat shock protein 70 (Hsp70) and heat shock protein 90 (Hsp90). This interaction requires the tetratricopeptide repeat (TPR) domain of PP5, which binds to an EEVD motif at the extreme C termini of cytosolic Hsp70 and Hsp90 isoforms. In addition to bringing PP5 into proximity with chaperone-bound substrates, this interaction also relieves autoinhibition in PP5’s catalytic domain, promoting its phosphatase activity. To better understand the molecular determinants of this process, we screened a large, pentapeptide library for binding to PP5. This screen identified the amino acid preferences at each position, which we validated by showing that the optimal sequences bind 4- to 7-fold tighter than the natural EEVD motifs and stimulate PP5’s enzymatic activity. The enhanced affinity for PP5’s TPR domain was confirmed using a protein-adaptive differential scanning fluorimetry assay. Using this increased knowledge of structure-activity relationships, we re-examined affinity proteomics results to look for potential EEVD-like motifs in the C termini of known PP5-binding partners. This search identified elongator acetyltransferase complex subunit 1 (IKBKAP) as a putative partner, and indeed, we found that its C-terminal sequence, LSLLD, binds directly to PP5’s TPR domain *in vitro*. Consistent with this idea, mutation of elongator acetyltransferase complex subunit 1’s terminal aspartate was sufficient to interrupt the interaction with PP5 *in vitro* and in cells. Together, these findings reveal the sequence preferences of PP5’s TPR domain and expand the scope of PP5’s functions to include chaperone-independent complexes.

Most protein phosphatases, such as PPP1/2 and calcineurin, work as part of multiprotein complexes composed of a catalytic subunit bound to a combination of targeting, scaffolding, and/or regulatory subunits that, together, tune substrate selection and phosphatase activity (1, 2). However, among this family of enzymes, protein phosphatase 5 (PP5) is unique because it lacks any of the classic partners. Rather, the function of PP5 is regulated by its association with the molecular chaperones, Hsp70 and Hsp90 (3). In these complexes, the chaperones act as adapters, binding PP5 and bringing it in proximity with chaperone-bound substrates (4). Because chaperones interact with a wide range of important signaling proteins (5), there is interest in better understanding how PP5’s activity is shaped by its chaperone interactions.

PP5 is a 58 kDa protein composed of a catalytic domain and a tetratricopeptide repeat (TPR) domain. Structural studies have shown that apo-PP5 resides in an autoinhibited state in which its TPR domain is disordered, and an α-J helix is positioned to block access to the phosphatase active site (6, 7). When Hsp70s or Hsp90s bind to PP5’s TPR domain, they induce a conformational change that relieves this autoinhibition (6, 8). In this way, the chaperones seem to ensure that PP5’s activity is stimulated when it is in proximity with chaperone-bound substrates.

The major cytosolic Hsp70s (HSPA1A and HSPA8) and Hsp90s (HSP90AA and HSP90AB) have a conserved EEVD motif at their extreme C termini. Specifically, the Hsp70 isoforms end in the pentapeptide sequence, IEEVD, while the Hsp90s have a terminal MEEVD sequence. These M/IEEVD sequences are thought to make extensive, electrostatic interactions with cationic residues in PP5’s TPR domain, termed the “carboxylate clamp” (9, 10). In humans, there are ∼30 other TPR-domain proteins with carboxylate clamps (11, 12), including the E3 ubiquitin ligase CHIP. Recently, the absolute specificity of CHIP’s TPR domain was determined by measuring its binding to EEVD-like peptides (13), revealing a strong and unexpected preference for proline at the penultimate residue and tryptophan residues instead of the two glutamic acids. Guided by these structure–activity relationships, tight binding sequences were discovered in the C termini of other (*e.g.*, nonchaperone) proteins; for example, CHIP was found to bind a proteoform of the microtubule-associated protein tau through an EEVD-like sequence (13). This noncanonical interaction also seems to be biologically important because CHIP selectively promotes the turnover of this proteoform *in vivo* (14, 15, 16). These studies suggest the possibility that other carboxylate clamp containing TPR cochaperones, such as PP5, might also have unanticipated sequence preferences, allowing them to identify C-terminal sequences in nonchaperone proteins. If this is indeed a broader property of TPR cochaperones, then the biology of these proteins seems likely to extend beyond Hsp70- and Hsp90-bound proteins.

Here, we explore the sequence preferences of PP5’s TPR domain. Using fluorescence polarization (FP), we first confirm (3) that PP5 prefers the MEEVD sequence in Hsp90s over the IEEVD sequence in Hsp70s (by about 9-fold). Then, we use truncations, alanine scans, and a positional scanning synthetic combinatorial library (PSSCL) to explore the molecular determinants of binding to PP5’s TPR domain. These experiments reveal that PP5 has sequence preferences that are very different from CHIP. To confirm this idea, we created peptides composed of the optimal amino acids at each position, such as WEEVD and WDDVD, showing that they bind up to 7-fold better to PP5 than the natural sequence and activate phosphatase activity. Finally, we use this knowledge of PP5’s preferences to search for potential EEVD-like sequences in known PP5-binding partners. We find that elongator acetyltransferase complex subunit 1 (ELP1), a core component of the elongator complex, has a C-terminal EEVD-like motif that binds directly to PP5 *in vitro* and in cells. In phosphatase assays, the C-terminal pentapeptide from ELP1 was able to stimulate PP5 activity by more than 2-fold. We suggest that this mechanism allows some phosphatase substrates, such as ELP1, to interact directly with PP5, eschewing the use of the chaperones as adapters.

## Results

### PP5 shows a preference for binding to the C-terminal MEEVD motif of Hsp90

**Figure 1:**
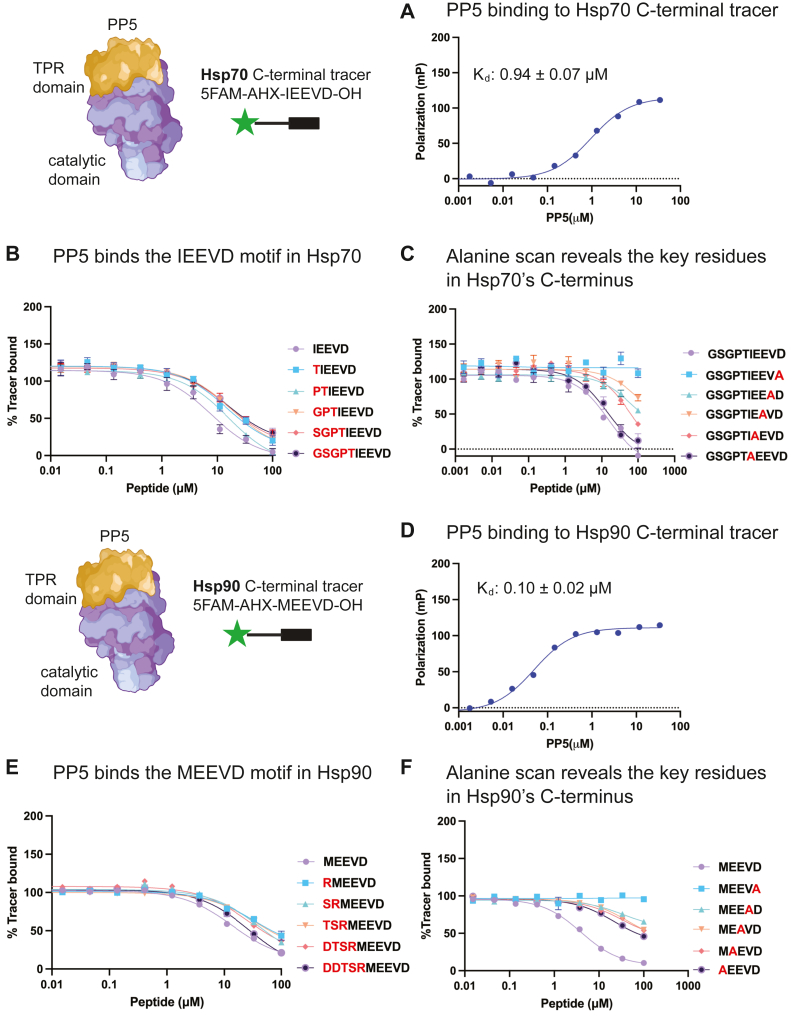
**PP5 shows a preference for binding the last five amino acids of Hsp90.***A*, binding of an Hsp70 tracer (5FAM-AHX-IEEVD) to PP5. Results are representative of three biological replicates. Each experiment was performed in technical triplicate, and the calculated Kd value is an average of all replicates (n = 9). Error bars represent standard error of the mean (SD). Some bars are smaller than the symbols. *B*, competition FP studies using Hsp70-derived acetylated 10mer peptides, revealing the key role for the last five amino acids. *C*, alanine scan of the first five amino acids in the context of acetylated 10mer peptides. *D*, binding of an Hsp90 tracer (5FAM-AHX-MEEVD) to PP5. Results are representative of three biological replicates. Each experiment was performed in technical triplicate, and the calculated *K*_*d*_ value is an average of all experiments (n = 9). Error bars represent SD, and some bars are smaller than the symbols. *E*, competition FP studies using Hsp90-derived acetylated 10mer peptides, revealing the key role for the last five amino acids. *F*, alanine scan of Hsp90 MEEVD sequence. For the experiments in panels *B*, *C*, *E*, and *F*, the studies were performed in technical quadruplicate (n = 4), and the results are representative of two independent experiments. Error bars represent standard deviation (SD), and some bars are smaller than the symbols. 5FAM, 5-carboxyfluorescein; AHX, aminohexanoic acid; FP, fluorescence polarization; Hsp70, heat shock protein 70; Hsp90, heat shock protein 90; PP5, protein phosphatase 5; TPR, tetratricopeptide repeat.

The TPR domain of PP5 is known to interact with the extreme C-terminal residues of Hsp90s and Hsp70s (3, 17). To confirm this finding and build an assay for exploring the sequence determinants of the interaction, we synthesized fluorescently labeled tracers composed of the last five residues from either the Hsp90 sequence [5-carboxyfluorescein [5FAM]-aminohexanoic acid (AHX)-MEEVD] or the Hsp70 sequence (5FAM-AHX-IEEVD). We then measured binding of these tracers to purified, human full-length PP5 using FP. The FP results indicate that the *K*_*d*_ of PP5 for the Hsp70 IEEVD tracer is 0.941 ± 0.070 μM (Fig. 1*A*), whereas the *K*_*d*_ for the Hsp90 MEEVD tracer is 0.102 ± 0.020 μM (Fig. 1*D*). These values match well with previously reported affinity constants (3), establishing a benchmark for further studies. Then, to investigate the influence of upstream residues on binding, we synthesized acetylated peptides corresponding to the last ten amino acids of Hsp70 (HSPA1A; Ac-GSGPTIEEVD-OH) or Hsp90 (HSP90AA; Ac-DDTSRMEEVD-OH), along with truncations in which the N-terminal residues were systematically removed. Using these peptides in FP competition assays, we found that none of the N-terminal residues were important for binding in either the Hsp70 (Fig. 1*B*) or Hsp90 (Fig. 1*E*) sequences. For example, the Hsp70-derived IEEVD pentapeptide bound with approximately the same *K*_*i*_ value (∼8 μM) as the 10mer (∼15 μM), while the Hsp90-derived MEEVD pentapeptide had a *K*_*i*_ value (∼14 μM) that is nearly equivalent to the 10mer (∼27 μM). These findings suggest that most of the binding energy of this interaction is due to the last five amino acids of the C termini. Next, we explored which residues within the pentapeptide contribute to affinity using alanine replacements. This experiment has been reported for Hsp90 10mer peptides, so here we focused on just the pentapeptide. In competition FP studies, we found that the last amino acid, aspartic acid, was essential, for either the Hsp70 (Fig. 1*C*) or Hsp90 (Fig. 1*F*) peptides. As a convention, we term this terminal residue, position 1 (P1), and then count back the other residues toward the N terminus as P2, P3, *etc.* In this nomenclature, the alanine scan also revealed that the identity of the P2, P3, and P4 residues are important for Hsp70 peptide binding (Fig. 1*C*). For example, mutating the P3 glutamic acid to an alanine removed at least 80% of the affinity (*K*_*d*_ > 100 μM). In contrast, mutation of the P5 isoleucine residue had no effect (*K*_*i*_ ∼ 13.6 μM). In the case of Hsp90 sequences, all of the positions P2 through P5 seemed to be equally important (Fig. 1*F*). Thus, PP5 primarily makes contact with the last five amino acids of the chaperones and, to somewhat varying extents, each of those positions contributes to binding.

### Determination of the absolute specificity of the PP5 TPR domain using an EEVD-like peptide library

**Figure 2:**
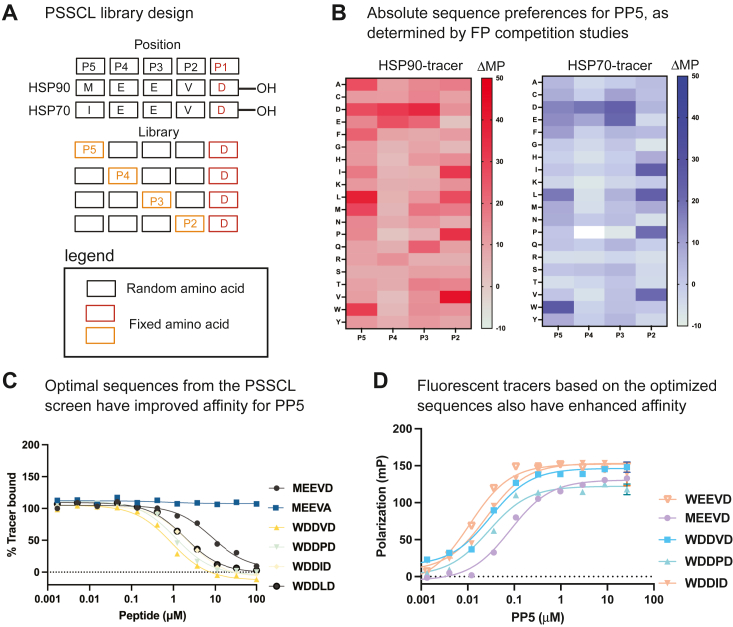
**Characterization of the sequence preferences of PP5’s TPR domain.***A*, design of the EEVD-like PSSCL collection. Each position in the C-terminal sequence is assigned a position number (P1, P2, *etc.*). The sequences of Hsp70 and Hsp90 are shown. The P1 aspartic acid was held constant in all pentapeptide pools. See the text for details. *B*, results of the competition FP studies using either the Hsp90 tracer (*left*) or the Hsp70 tracer (*right*). The heat map shows the relative effect on tracer binding. *C*, optimized peptides bind tighter than MEEVD to PP5 in competition FP experiments. Experiments were performed in technical quadruplicate (n = 4), and the results are representative of two independent experiments. Error bars represent standard deviation. *D*, fluorescent tracers with the optimized sequences bind tighter to PP5 in direct binding experiments. Experiments were performed in technical quadruplicate (n = 4), and the results are representative of two independent experiments. Error bars represent SD. Hsp70, heat shock protein 70; Hsp90, heat shock protein 90; FP, fluorescence polarization; PP5, protein phosphatase 5; PSSCL, positional scanning synthetic combinatorial library; TPR, tetratricopeptide repeat.

To more broadly explore PP5’s sequence preferences, we screened an “EEVD-like library” synthesized using a PSSCL approach (18). Briefly, this library is composed of 80 pools of acetylated pentapeptides, generated by automated solid-phase peptide synthesis. In each pool, the P2, P3, P4, and P5 positions were set to the 20 natural amino acids, and the remaining sites were then randomized (Fig. 2*A*), while the P1 aspartic acid was held constant. Altogether, this library samples around 640,000 possible EEVD-like sequences (13). To screen the library for binding to PP5, we employed two parallel FP competition screens, using either the Hsp90 tracer or the Hsp70 tracer (Fig. 2*B*). Satisfyingly, the results of the two screens agreed well with each other, although, as expected, the dynamic range was better using the tighter binding Hsp90 tracer. When we compiled the results of these competition studies, we find that PP5 prefers a subset of bulky residues, especially tryptophan, at the P5 position (Fig. 2*B*). At P4 and P3, there is a strong preference for the anionic residues, glutamic and aspartic acid, while at the P2 position, a subset of small, nonpolar amino acids (*e.g.*, valine, proline, isoleucine, and leucine) are preferred. These preferences are, at some positions, dramatically different from those of CHIP (13, 19), suggesting that TPR cochaperones have partially distinct requirements for tight binding to C-terminal peptides (see the Discussion).

To test these predictions, we synthesized acetylated pentapeptides corresponding to the optimal amino acids at each position. We considered this experiment to be important because the PSSCL approach does not fully account for potential effects of changing multiple residues simultaneously, so a pentapeptide composed of the best amino acid at each position might not be considerably better than the natural sequences. Using competition FP experiments, we confirmed that the optimized peptides, WEEVD, WDDVD, WDDPD, WDDID, and WDDLD, all have tighter affinity than the natural MEEVD (Fig. 2*C*). Also, the fact that these pentapeptides compete with the Hsp90 tracer further reinforces the idea that they bind to the known cleft in PP5’s TPR domain. To confirm the tighter binding and provide new tools for studying PP5, we synthesized fluorescent tracers of the best sequences (5FAM-AHX-WEEVD, 5FAM-AHX-WDDVD, 5FAM-AHX-WDDPD, and 5FAM-AHX-WDDID). Consistent with the model, each of these tracers bound tighter than the natural sequence, with WDDVD having an approximately 6-fold improved affinity (*K*_*d*_ = 0.017 ± 0.010 μM; Fig. 2*D*) and WEEVD having approximately 7-fold better binding (*K*_*d*_ 0.015 ± 0.010 μM Fig. 2*D*). Thus, the natural EEVD motifs in Hsp70s and Hsp90s are not optimized for binding to PP5; tighter binding C termini are possible.

### Development of a protein-adaptive differential scanning fluorimetry assay to study binding to PP5

**Figure 3:**
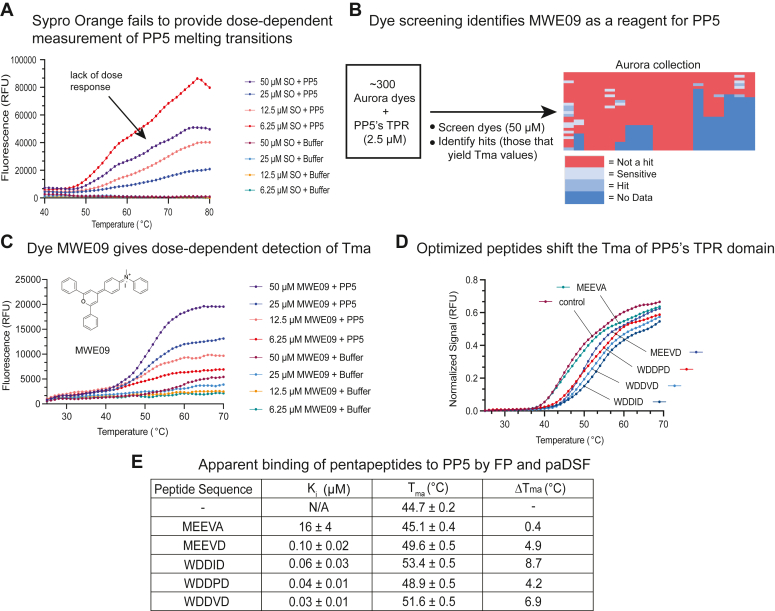
**Development of a protein-adaptive differential scanning fluorimetry (paDSF) assay and confirmation that optimized pentapeptides bind tighter to PP5’s TPR domain.***A*, the dose dependence of Sypro Orange (SO) detection of melting transitions in PP5. Note the strong decrease in signal at 25 μM SO. Results are representative of experiments performed three times. *B*, schematic depiction of the dye screening workflow to identify alternative reagents for paDSF experiments. A screen of 300+ Aurora dyes against PP5’s TPR domain yielded multiple dose-dependent “hits”, including MWE09. Results are representative of experiments performed in duplicate. *C*, dose response of MWE09 detection of melting transitions in PP5. The chemical structure of MWE09 is shown. Results are representative of experiments performed in triplicate. *D,* addition of optimized pentapeptides causes a rightward shift in the T_ma_ values of PP5’s TPR domain. PP5 TPR domain (2.5 μM), MWE09 (50 μM), and pentapeptides (10 μM). Results are the average of experiments performed in quadruplicate. *E*, summary of the calculated Ki (from competition FP studies on full-length PP5) and deltaT_ma_ values (from paDSF using PP5’s TPR domain), showing qualitative correlation. FP, fluorescence polarization; PP5, phosphatase 5; TPR, tetratricopeptide repeat.

To provide a secondary binding assay, we sought to develop a protein-adaptive differential scanning fluorimetry (paDSF) method for PP5. In classical DSF, a solvatochromatic dye, Sypro Orange (SO), is used to monitor thermal unfolding of a protein. Typically, the fluorescence of the dye is plotted against temperature to calculate an apparent melting transition (T_ma_) (20). However, SO is not compatible with ∼60% of proteins (21) and in early experiments, we found that this dye produces artifacts in the presence of PP5 (Fig. 3*A*), likely due to co-aggregation. Recently, paDSF was developed, in part, to solve these types of technical issues (21). In psDSF, a library of fluorescent probes (termed the Aurora collection) is first screened to identify a reagent that monitors the intended melting transition. Essentially, this workflow replaces SO for a dye with better behavior under the experimental conditions (*e.g.*, buffer, protein, *etc.*). To create a paDSF assay for PP5, we first screened PP5’s TPR domain (2.5 μM) with each of 300+ Aurora dyes (50 μM) in 384-well plates and then used DSFworld (22) to fit the results and select potential “hits” that yield a T_ma_ value (Fig. 3*B*). From this screen, we identified MWE09 as a useful probe for PP5’s TPR domain, because it yielded a T_ma_ value (44.7 °C) that roughly approximates the literature value of ∼35 to 40 °C from circular dichroism under different buffer conditions (7). Using this paDSF protocol, we then measured binding of PP5’s TPR domain to the EEVD-like pentapeptides (10 μM) by quantifying a change in the apparent melting temperature (ΔT_ma_). We found that, as expected, addition of MEEVD shifted the T_ma_ value of PP5’s TPR domain to be 49.6 °C (Fig. 3*D*), which represents a ΔT_ma_ value of +4.9 °C. Moreover, the MEEVA peptide, in which the key aspartic acid is mutated, failed to bind (T_ma_ value 45.1 °C; ΔT_ma_ +0.4 °C). When we also tested a subset of the optimized pentapeptides, we satisfyingly noted that their ΔT_ma_ values qualitatively align with the relative *K*_*i*_ values calculated from FP studies (Fig. 3*E*), supporting the idea that these EEVD-like sequences bind to PP5’s TPR domain. More broadly, we hope that this paDSF protocol could enable more studies of PP5’s interactions.

### The phosphatase activity of PP5 is activated by EEVD-like peptide binding

**Figure 4:**
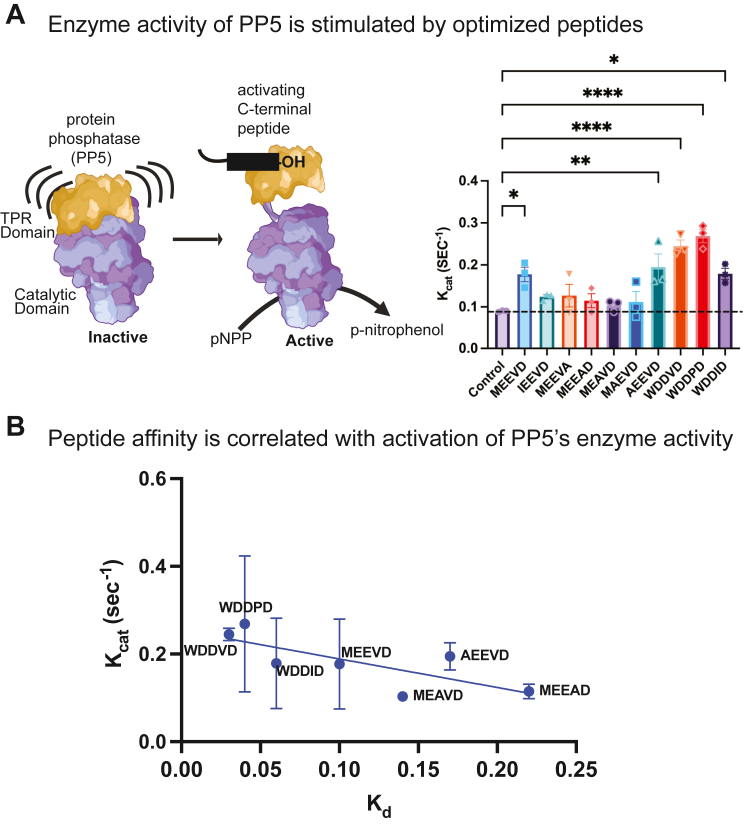
**Optimized pentapeptides stimulate the phosphatase activity of PP5.***A*, measurement of PP5 activity using pNPP substrate in a colorimetric assay. Results are the average of three independent experiments performed in triplicate each (n = 9). Error bars represent SD. Statistical significance was determined by one-way ANOVA, (∗*p* < 0.05, ∗∗*p* < 0.01, ∗∗∗∗*p* < 0.001). *B*, correlation plot between the measured *k*_*cat*_ and *K*_*m*_ values from the enzyme assays and *K*_*d*_ values from FP, showing that these features correlate with each other. FP, fluorescence polarization; pNPP, p-nitrophenyl phosphate; PP5, protein phosphatase 5.

Binding of either EEVD peptides or polyunsaturated lipids, such as arachidonic acid, is known to cause a conformation change that stimulates PP5’s phosphatase activity (3, 23, 24). Thus, expected that the optimized pentapeptides might also stimulate PP5’s phosphatase activity, if they engage with the key residues involved in this allosteric process. To test these ideas, we turned to a commonly used p-nitrophenyl phosphate (pNPP) colorimetric assay to measure PP5’s catalytic activity *in vitro*. As expected, treatment of PP5 with peptides of the natural EEVD sequences or a subset of the optimized peptides (*e.g.*, WDDVD, WDDPD, and WDDID) resulted in enhanced turnover (Fig. 4*A*). As expected from previous studies (24), this effect was largely observed in an improved *k*_*cat*_, with less pronounced impact on *K*_*m*_ (Fig. S1*A*). In fact, some of the optimized peptides modestly weakened the *K*_*m*_ value. We also found that the optimized peptides, such as WDDVD, had a somewhat greater stimulatory effect on *k*_*cat*_ (0.24 ± 0.01 s^−1^; 2.5-fold) than the natural MEEVD sequence (0.17 ± 0.01 s^−1^; 2-fold). Indeed, we noticed a roughly linear relationship between the peptide’s *K*_*d*_ values and their impact on *k*_*cat*_ (Fig. 4*B*), suggesting that binding and activation of PP5 are correlated. As expected, the overall effect on *k*_*cat*_/*K*_*m*_ was less pronounced (Fig. S1*B*). Together, these findings seem consistent with the prevailing idea that peptide binding to PP5’s TPR domain causes a disorder-to-order transition (8, 25, 26, 27), likely coupling binding to conformational transitions in the adjacent catalytic domain and increased turnover.

### ELP1 contains an EEVD-like motif and binds directly to PP5 in a chaperone-independent mechanism

**Figure 5:**
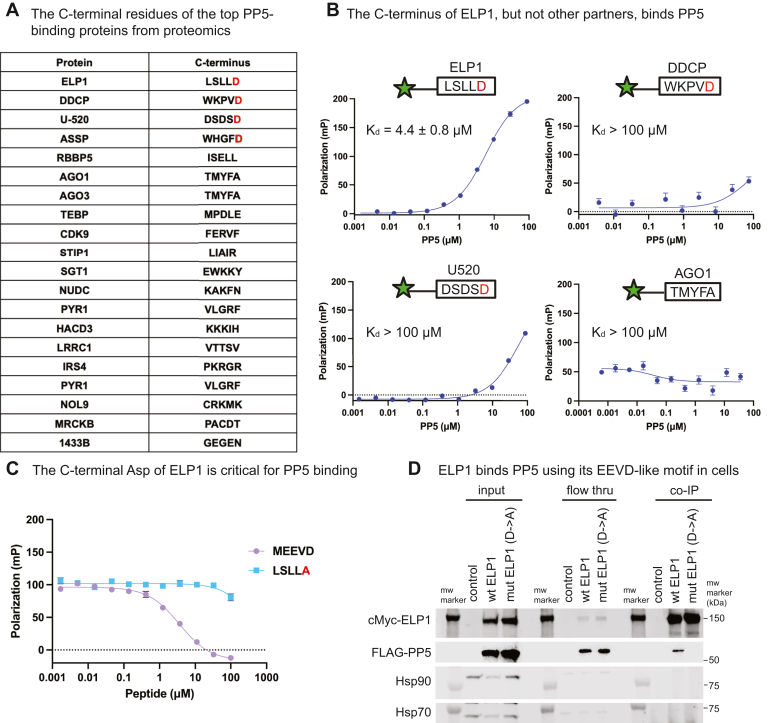
**Evaluation of the C termini of known PP5 partners reveals ELP1 as having an EEVD-like motif that directly binds PP5**. *A*, list of the C-terminal five amino acids for each of the top 20 partners of PP5 from a published interactome study. *B*, fluorescent tracer with the ELP1 sequence, LSLLD, but not the other sequences, binds to PP5. Results are representative of experiments performed three times in triplicate each. Error bars represent SD, and some bars are smaller than the symbols. Calculated *K*_*d*_ values are from all three experiments (n = 9). *C*, competition FP experiments showing that LSLLD competes with Hsp90’s MEEVD for binding and that the P1 aspartate is required. Results are representative of three independent experiments performed in technical triplicates. Error bars represent SD, and some bars are smaller than the symbols. *D*, HEK293T cells were transiently transfected with cMyc-ELP1 and FLAG-PP5. Co-immunoprecipitation of cMyc-ELP1 was followed by Western blots for FLAG, Hsp90, and Hsp70. Results are representative of experiments performed in duplicate (see Fig. S2).

Previous affinity proteomics studies have identified the proteins that interact with PP5 (28). This list seems likely to include substrates that are recruited by Hsp70 and Hsp90 in the canonical mechanism. However, we reasoned that a subset of these PP5 partners might also have their own EEVD-like motifs, therefore binding in a chaperone-independent way. To explore this hypothesis for PP5, we first examined the C-terminal sequences in the top 20 proteins identified in the reported PP5 interactome (Fig. 5*A*). We noticed that the top four binding partners have an aspartic acid at their terminal P1 position, immediately suggesting that they could indeed be direct partners. Of these sequences, LSLLD (from ELP1) seemed most promising based on the observed structure-activity relationships (see Fig. 2*B*). Specifically, leucine was identified as one of the preferred residues at P2 and P5, and there are no clear conflicting residues at the other positions (*e.g.*, serine is neutral at P4 and leucine is neutral at P3). The C-terminal sequence WKPVD from the protein DDCP was also interesting, given the preferred residue identity at P5 (tryptophan) and P2 (valine); however, the P3 proline seemed likely to cause conflicts. To test these predictions, we synthesized fluorescent tracers of four of the targets: ELP1, DDCP, U520, and ASSP, along with a negative control, AGO1 (which is a PP5 partner but lacks the essential P1 aspartic acid). Using FP experiments, we found that only the ELP1 tracer (5FAM-AHX-LSLLD) interacts with PP5, with a *K*_*d*_ of 4.4 ± 0.8 μM (Fig. 5*B*), whereas the other tracers did not bind with appreciable affinity (*K*_*d*_ > 100 μM). We could not determine an answer for ASSP, as the corresponding tracer tended to aggregate under the FP conditions.

Using the ELP1 tracer, we confirmed in competition studies that unlabeled MEEVD peptide could compete for binding (Fig. 5*C*), again suggesting that the EEVD-like sequence of ELP1 competes for the shared site on PP5’s TPR domain. Moreover, mutating the C-terminal aspartic acid of the ELP1 sequence to alanine (LSLLA) completely suppressed binding in competition studies (Fig. 5*C*).

To ascertain whether ELP1 binds to PP5 in cells, we transfected HEK293T cells with cMyc-ELP1 and FLAG-PP5 and performed immunoprecipitations. The results confirm the reported mass spectrometry findings (28), showing that ELP1 binds PP5 (Figs. 5*D* and S2). Importantly, we find that this complex did not include substantial amounts of Hsp90 or Hsp70, consistent with the fact that only one EEVD or EEVD-like motif can be bound to PP5 at once. Moreover, mutating only the terminal aspartic acid in ELP1 (mut ELP1; ending in LSLLA) fully prevented the interaction with PP5 (Figs. 5*D* and S2). Thus, ELP1 uses a chaperone-independent mechanism to directly bind to PP5 through its EEVD-like, C-terminal motif.

**Figure 6:**
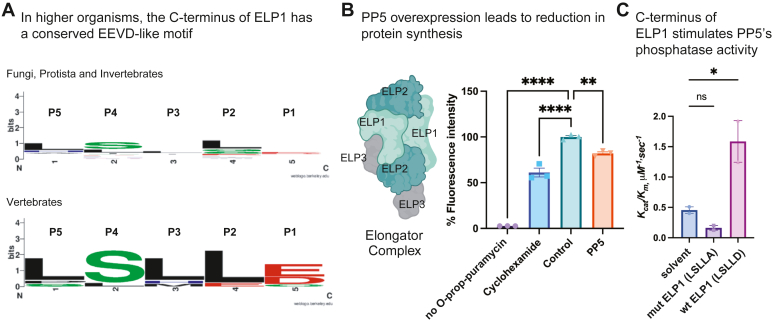
**ELP1 has a conserved EEVD-like motif that seems to coordinate with PP5 to regulate its functions.***A*, LogoPlot of the C-terminal pentapeptides from ELP1 in both lower organisms and vertebrates (Fig. S3). Note the conserved acidic residue in the P1 position and the small, aliphatic residues in P2. *B*, translation efficiency assays in HEK293T cells treated with DMSO (control), cycloheximide (positive control), or overexpressing PP5. Results are the average of experiments performed in three independent replicates. Error bars represent SD. Statistical significance was determined by one-way ANOVA (∗∗*p* < 0.01, ∗∗∗∗*p* < 0.0001). *C*, phosphatase assays show that the C-terminal pentapeptide from ELP1 (LSLLD; 100 μM) but not the alanine mutant (LSLLA; 100 μM) stimulates PP5. Solvent is DMSO (1%). Results are the average of independent duplicates and error bars represent SD. Statistical significance was determined by one-way ANOVA (∗*p* < 0.05). ELP1, elongator acetyltransferase complex subunit 1; PP5, protein phosphatase 5.

We next examined the evolutionary conservation of ELP1’s C-terminal residues. Intriguingly, we observed no substantial preference for aspartate at the P1 position in a subset of lower organisms, such as fungi, protists, or invertebrates (Fig. S3), but in vertebrates (Fig. S3), the anionic residues, aspartate and glutamate, were enriched (Fig. 6*A*). This observation suggests that ELP1 might have evolved in vertebrates to recruit PP5 through an EEVD-like sequence. As an initial test of the functional relevance of such an interaction, we measured the effects of PP5 overexpression on global translation in HEK293T cells. As mentioned above, ELP1 is part of the elongator complex that plays important roles in multiple cellular processes (29), including translation (30). Moreover, phosphorylation of ELP1 is known to be required for this process (31), so we hypothesized that overexpression of PP5 might suppress translation. In HEK293T cells, we first confirmed that treatment with cycloheximide (50 μg/ml) produced the expected 40% decrease in translation when compared to the DMSO control (Fig. 6*B*). Then, we performed an overexpression of PP5 and found that this treatment led to an ∼25% decrease in translation (Fig. 6*B*). Although this simple, phenotypic experiment does not address whether the mechanism of PP5 is direct *versus* indirect, the results generally support a model in which PP5 is a putative regulator of ELP1. Finally, to test whether ELP1 binding, like chaperone binding, might stimulate PP5’s enzyme activity, we turned to the *in vitro* pNPP assays. Consistent with the model, treatment of PP5 with the LSLLD peptide stimulated enzyme activity, while the LSLLA control did not (Fig. 6*C*). In this case, both the *k*_*cat*_ and *K*_*m*_ were improved, such that the *k*_*cat*_/*K*_*m*_ of PP5 was improved more than 2-fold (from 0.49 μM^−1^sec^−1^ to 1.28 μM^−1^sec^−1^) by treatment with the ELP1 C-terminal pentapeptide.

**Figure 7:**
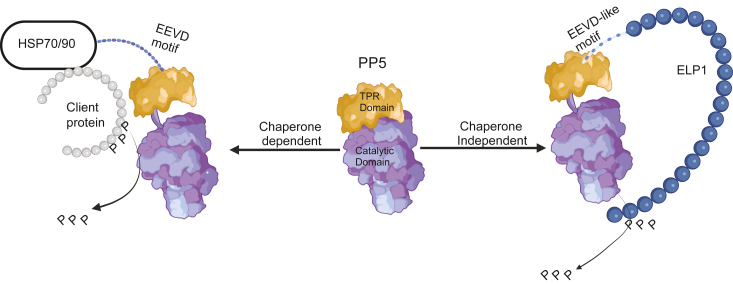
**ELP1 has a conserved C-terminal EEVD-like motif that binds PP5 in a chaperone-independent mechanism.** Model for the chaperone-dependent and chaperone-independent mechanisms of PP5 action. Autoinhibited PP5 is activated by binding to Hsp90 or Hsp70, bringing its activity in proximity to chaperone-bound clients. In the proposed pathway, certain proteins, such as ELP1, have EEVD-like motifs that bind directly to PP5, presumably directing phosphatase activity to itself and potentially nearby proteins. ELP1, elongator acetyltransferase complex subunit 1; Hsp70, heat shock protein 70; Hsp90, heat shock protein 90; PP5, protein phosphatase 5.

Taken together, we suggest that PP5 uses two distinct mechanisms for selecting its substrates. In the canonical mechanism, it is recruited to substrates through Hsp70 and Hsp90 (Fig. 7; left). Here, we provide evidence that PP5 also binds directly to some substrates, such as ELP1, that contain a C-terminal EEVD-like motif (Fig. 7; right). This possibility expands PP5 functions to include chaperone-independent substrates.

## Discussion

The interactions of cytosolic molecular chaperones, Hsp70 and Hsp90, with TPR-domain containing cochaperones, such as CHIP and PP5, are important for protein homeostasis (12). Each of these complexes involves binding of the chaperone’s EEVD motif to the carboxylate clamp of the TPR domains (32). However, recent work has shown that some nonchaperone proteins have EEVD-like motifs in their C termini, allowing them to bind directly to CHIP (13). This finding inspired us to consider the possibility that other TPR-domain cochaperones, such as PP5, might also have chaperone-independent partners, a finding that could substantially expand their known roles.

Here, we focused on PP5 as an important TPR-domain cochaperone because it has been associated with numerous important pathways (4, 33, 34, 35), and unlike the other members of the protein phosphatase family, it does not have associated targeting or regulatory subunits. Rather, in the canonical model, Hsp70 and Hsp90 recruit PP5 through their EEVD motifs, locally activating phosphatase activity in the proximity of chaperone-bound substrates (see Fig. 7; left). To probe the molecular determinants of this interaction, we used biophysical experiments to show that PP5 prefers the Hsp90 sequence (MEEVD) over the Hsp70 sequence (IEEVD). Indeed, PP5 has been shown to collaborate with Hsp90 in functional complexes (34). Using a PSSCL-derived pentapeptide library (13), we then determined the absolute sequence preferences of PP5 and confirmed that peptides composed of the optimized amino acid at each position, such as WEEVD, WDDVD, WDDPD, and WDDID, bind up to 4- to 7-fold tighter than the natural sequences (see Fig. 2). Combined with phosphatase activity assays, this search also allowed us to show that binding affinity is roughly correlated with enzyme activation (see Fig. 4). This finding is consistent with structural and biochemical studies (8, 27, 36), which suggest that ligand binding to PP5’s TPR domain leads to a disorder-to-order transition and a corresponding conformational change that relieves PP5 autoinhibition. This autoinhibition mechanism is likely important for PP5 function, as it ensures that the enzyme activity is low in the apo state. Yet, exploration of the sequence determinants of this activation process revealed that the natural EEVD peptides, as reported in the literature (3, 24), primarily stimulate *k*_*cat*_, with little effect on *K*_*m*_. The optimized peptides similarly activated *k*_*cat*_ but tended to weaken *K*_*m*_. Only the ELP1-derived peptide promoted both values and enhanced *k*_*cat*_/*K*_*m*_ by more than 2-fold (see Fig. 6*C*). The structural basis for these differences is unclear, as is any potential functional relevance.

A goal of this study was to reveal the sequence preferences of PP5’s TPR domain and compare them to other TPR domains. Interestingly, we find that the PP5 preferences are dramatically different from those of CHIP’s TPR domain (13). For example, CHIP seems to prefer bulky residues, such as tryptophan, at P3 and P4, whereas PP5 strongly prefers glutamate or aspartate at those same sites (see Fig. 2*B*). Thus, EEVD-like sequences that bind CHIP are unlikely to be good partners for PP5, suggesting divergence of the complexes. Instead, PP5’s preferences are more similar to those of another, reported EEVD-binding partner, DnaJB4 (19). DnaJB4 is a member of a class of Hsp70 cochaperones that binds to the C-terminal IEEVD motif (37, 38). Members of this cochaperone family do not have a TPR domain, and instead, they bind to the EEVD motif using a beta-sheet rich domain (39, 40). Given the stark structural differences between this domain and the TPR domains, it is interesting that PP5 and DnaJB4 have such similar sequence preferences; for example, they both prefer glutamate or aspartate at P3 and P4. Thus, we envision that PP5 and DnaJB4 might compete for binding to the IEEVD motifs in cytoplasmic Hsp70s, potentially helping to partition the chaperones into a variety of specific complexes. However, DnaJB4 does not require the P1 aspartate that is essential for PP5 binding, so there are also significant differences. These differences are also interesting, as they could be biologically important, and moreover, they might be leveraged to rationally build selective inhibitors of chaperone protein–protein interactions (41).

Using our knowledge of PP5 sequence preferences, we searched for potential chaperone-independent partners of PP5 by looking for C-terminal EEVD-like sequences in published affinity proteomics datasets (28). This search and subsequent biochemical studies revealed that ELP1 has a C-terminal EEVD-like motif (LSLLD) that binds to PP5’s TPR domain with modest affinity (*K*_*d*_ ∼ 4 μM) (see Fig. 5*B*). In cells, this binding is independent of chaperones and is completely dependent on the C-terminal aspartate (see Fig. 5*D*). Phosphorylation of ELP1 has a strong effect on its function (30, 31), suggesting that interactions with PP5 might be important in its regulation, which is an idea that is crudely supported by the effects of PP5 overexpression on global translation (see Fig. 6*B*). Finally, the C-terminal pentapeptide of ELP1, LSLLD, was sufficient to activate the enzyme functions of PP5 *in vitro* (Fig. 6*C*), suggesting that ELP1 could locally promote dephosphorylation. More work is needed to better understand the functional and mechanistic relationship between ELP1 and PP5. Here, we instead focus on the mystery of why the interaction between ELP1 and PP5 needs to be chaperone independent? One possibility is that ELP1 phosphorylation occurs within the context of the large multiprotein elongator complex (see schematic in Fig. 6*B*), which might preclude interactions with chaperones that prefer binding to unfolded regions. Another compelling question is whether other proteins, besides ELP1, might have EEVD-like motifs that bind directly to PP5. We failed to find interactions with the C termini of the other candidates, DDCP or U520 (see Fig. 5*B*), but this result does not preclude the idea that PP5’s functions could be further expanded.

## Experimental procedures

### Protein expression and purification

Human PP5 was expressed from a pMCSG7 vector with an N-terminal His-tag and TEV cleavage site in *Escherichia coli* BL21 (DE3) cells. Liter cultures of terrific broth were grown at 37 °C until an *A*_600_ of 0.6. Then, cultures were cooled to 18 °C before induction with IPTG (final concentration of 1 mM) and grown overnight. Cells were harvested by centrifugation (4000*g*, 10 min, 4 °C). For protein purification, cell pellets were resuspended in His-binding buffer (50 mM Tris, 10 mM imidazole, and 500 mM NaCl, pH 8) supplemented with Roche cOmplete protease inhibitor cocktail. Cells were lysed by sonication, pelleted by centrifugation, and the supernatant applied to Ni-NTA His-Bind Resin (Novagen). The resin was washed with His-binding buffer, followed by His-washing buffer (50 mM Tris, 30 mM imidazole, and 300 mM NaCl, pH 8). The protein was then removed from the resin using His-elution buffer (50 mM Tris, 300 mM imidazole, and 300 mM NaCl, pH 8). The N-terminal His-tag was removed using TEV protease (1 μM overnight at 4 °C), and the sample was purified by size-exclusion chromatography using a XK 16/100 Superdex 200 column (GE Healthcare Life Sciences) in a 50 mM Hepes and 10 mM NaCl, pH 7.4 buffer. A similar procedure was used to purify the isolated PP5’s TPR domain (residues 19–146). Proteins were stored at −80 °C in 5% glycerol until use.

### Fluorescence polarization

#### Saturation binding

Fluorescent peptides corresponding to the C-termini Hsp70 and Hsp90 were custom synthesized by GenScript with an N-terminal 5FAM linked *via* a 6-carbon spacer (AHX). These probes were stored as 10 mM DMSO stocks at −30 °C. Prior to use, tracer solutions were diluted in assay buffer (50 mM Hepes, 75 mM NaCl, and 0.001% Triton X-100, pH 7.4) to a working concentration of 40 nM. Each well received PP5 (5 μl) from a 3-fold dilution series prepared using the assay buffer. Subsequently, tracer (9 μl of 40 nM) was added to each well, resulting in a final concentration of 20 nM and a total assay volume of 18 μl. The plate was shielded from light and allowed to incubate at room temperature for 15 min, a duration determined to reach equilibrium during assay development. All experiments were conducted in 384-well, black, low-volume, round-bottom plates (Corning; catalog number = 4511). FP values, measured in millipolarization units, were recorded with a Molecular Devices Spectramax M5 plate reader at an excitation wavelength of 485 nm and an emission wavelength of 530 nm. Data analysis was carried out using GraphPad Prism 6. Similar methods were used to measure the affinity of other labeled tracer peptides.

#### Competition studies

For competition studies, each well had PP5 protein at a concentration equivalent to the Kd, and the tracer peptide concentration was 20 nM. Samples were incubated for 5 min prior to measurements. Results were analyzed as above.

### paDSF

#### Dye screens

Purified PP5 was screened against the Aurora collection of dyes, using methods that have been previously reported (21). Briefly, dye plates were prepared by transferring 250 nl of a 5 mM DMSO stocks to white, low-volume 384-well qPCR plate (Axygen PCR-284-LC480WNFBC) using an Echo 650 Acoustic Dispenser. Then, screening buffer (20 μl; 50 mM Hepes, 10 mM NaCl, pH 7.4) was transferred into each well of the dye plate using a E100 ClipTip p125 Matrix Pipette (Thermo Fisher #4671040BT). The plate was briefly centrifuged in a salad spinner for 30 s to remove bubbles. Then, an Opentrons OT-2 liquid handling robot was used to transfer 8 μl into a fresh white plate (as above) containing 10 μl of PP5, resulting in final concentrations of 2.5 μM protein and 50 μM dye. An identical plate was prepared without protein to control for artifacts (22). This plate was sealed with an optically clear sealing film and read in an Analytik Jena 384G qTower qPCR using “up-down” mode heating from 25 ºC to 95 ºC in increments of 1 °C every 30 s. These data were analyzed using DSFworld (22). Promising “hits” (*e.g.*, those dyes that yielded single transitions) were retested in dose response (50–6.25 μM dye). For this screen, dye MWE09 was selected for its clear transitions and good signal:noise (see Results).

#### Peptide binding

DSF experiments were carried out as above, with MWE09 (50 μM), PP5’s TPR domain (2.5 μM), and peptide or DMSO (10 μM). Plates were incubated for 5 to 10 min prior to measurements to allow for peptide binding. T_ma_ values were calculated using DSFWorld.

### Phosphatase assays

To measure PP5’s enzyme activity, pNPP was used as substrate, as previously described (24, 42). Briefly, purified PP5 (1 μM) was incubated with peptides (100 μM) in 40 mM Hepes, 20 mM KCl, 5 mM MnCl_2_, and 1 mM DTT, pH 7.5 at 20 °C in 96-well microtiter plates (total volume 50 μl). Reactions were then initiated by addition of the pNPP substrate at concentrations from 0 to 60 mM. After 30 min, reactions were quenched with 2N NaOH (100 μl), and the release of p-nitrophenol was recorded at 410 nm (ε = 15,100 M − 1 cm−1) on a Molecular Devices M5 plate reader. Enzyme parameters (*e.g.*, *K*_*m*_, *k*_*cat*_) were calculated using the Michaelis–Menten equation in Graphpad Prism 6.

### Cell culture

HEK293T cells (ATCC) were cultured in Dulbecco’s Modified Eagle Medium supplemented with 10% fetal bovine serum and 1% penicillin/streptomycin. These cells were maintained at 37 °C/5% CO_2_ in a humidified incubator. Cells were negative for *mycoplasma* contamination (MicroSeq; ThermoFisher Scientific) at the start of the experiments, and they were used without further authentication.

### Western blotting

Adherent HEK293T cells were collected by scraping in ice-cold PBS, followed by centrifugation for 5 min at 300*g*. The resulting pellet was washed with PBS, then lysed directly in RIPA buffer (50 mM Tris pH 8.0, 150 mM NaCl, 1% Nonidet P40, 0.5% sodium deoxycholate, and 0.1% SDS) supplemented with Roche cOmplete protease inhibitor cocktail. Lysates were incubated for 30 min on ice and then centrifuged at 21,000*g* for 10 min at 4 °C. The soluble fraction was quantified by BCA assay and normalized to a concentration of 2 mg/ml, then mixed with 5X reducing Laemmli buffer, and denatured for 5 min at 95 °C. Protein (20 μg) was loaded onto a 7.5% mini-TGX StainFree gel and separated at 120V for 60 min. The gel was transferred to 0.2 μm nitrocellulose membranes using the Bio-Rad Trans-Blot Turbo system. Blots were blocked in Intercept TBS blocking buffer (LI-COR) for 30 min at room temperature and then incubated with primary antibody in blocking buffer overnight at 4 °C. The following day, blots were washed three times for 5 min each with TBS + 0.05% Tween-20 and then incubated with 1:10,000 secondary antibodies (LI-COR) for 1 h at room temperature. Finally, blots were washed for 3 × 5 min in TBS + 0.05% Tween-20 and imaged on a LI-COR Fc imaging system.

### Co-immunoprecipitation

HEK293T cells were seeded at a density of 500K cells/well in a 6-well plate (Corning 3335), grown overnight, and then transfected using Lipofectamine-3000 according to the manufacturer’s protocol. The following day, cells were harvested in ice-cold PBS, then lysed in ice-cold lysis buffer (25 mM Tris-HCl pH 7.4, 150 mM NaCl, 1 mM EDTA, 1% NP-40, and 5% glycerol, with Roche cOmplete protease inhibitor cocktail) by trituration followed by incubation on ice for 10 min. Lysates were centrifuged for 10 min at 21,000*g*, and the soluble fraction was harvested and quantified by BCA assay. An input sample was retained, and 200 μg of lysate was then diluted to a final volume of 500 μl in lysis buffer. Diluted lysate was added to 20 μl of anti-myc magnetic resin (Pierce cat. 88843) in a 1.5 ml low-binding tube (Eppendorf), then incubated at RT for 3 h with end-over-end rotation. Following incubation, beads were washed with 3 × 500 μl of lysis buffer. Proteins were eluted by heating to 95 °C in 50 μl of 1× Laemmli buffer for 5 min and processed for Western blotting as above.

### Translation assay

The global translation assay (Abcam; ab273286) was used to measure protein synthesis according to the manufacturer’s specifications. Briefly, in this method, O-propargyl-puromycin is used to trap actively translating ribosomes, followed by addition of a fluorescent Click probe, such that suppression of translational is measured as a decrease in fluorescence. HEK293T cells or HEK293T cells overexpressing FLAG-PP5 were cultured as above. Cells were incubated with the supplied protein label reagent and incubated for 24 h, after which the cells were washed with culture media and fixed/permeabilized for 15 min in the dark. Click reactions were carried out for 30 min, and the plate fluorescence measured at Ex/Em 494/521 nm in a SpectraMax M5 multimode plate reader.

## Data availability

All data are contained within the manuscript or shared upon request.

## Supporting information

This article contains supporting information (Figs. S1–S3).

## Conflict of interest

The authors declare no conflicts of interest with the contents of this article.

## References

[1] Kohn M. (2020). Turn and face the strange: a new view on phosphatases. ACS Cent. Sci..

[2] Cohen P. (1994). The discovery of protein phosphatases: from chaos and confusion to an understanding of their role in cell regulation and human disease. Bioessays.

[3] Connarn J.N., Assimon V.A., Reed R.A., Tse E., Southworth D.R., Zuiderweg E.R. (2014). The molecular chaperone Hsp70 activates protein phosphatase 5 (PP5) by binding the tetratricopeptide repeat (TPR) domain. J. Biol. Chem..

[4] Sager R.A., Dushukyan N., Woodford M., Mollapour M. (2020). Structure and function of the co-chaperone protein phosphatase 5 in cancer. Cell Stress Chaperones.

[5] Hartl F.U., Bracher A., Hayer-Hartl M. (2011). Molecular chaperones in protein folding and proteostasis. Nature.

[6] Kang H., Sayner S.L., Gross K.L., Russell L.C., Chinkers M. (2001). Identification of amino acids in the tetratricopeptide repeat and C-terminal domains of protein phosphatase 5 involved in autoinhibition and lipid activation. Biochemistry.

[7] Yang J., Roe S.M., Cliff M.J., Williams M.A., Ladbury J.E., Cohen P.T. (2005). Molecular basis for TPR domain-mediated regulation of protein phosphatase 5. EMBO J..

[8] Cliff M.J., Williams M.A., Brooke-Smith J., Barford D., Ladbury J.E. (2005). Molecular recognition *via* coupled folding and binding in a TPR domain. J. Mol. Biol..

[9] Freeman B.C., Myers M.P., Schumacher R., Morimoto R.I. (1995). Identification of a regulatory motif in Hsp70 that affects ATPase activity, substrate binding and interaction with HDJ-1. EMBO J..

[10] Scheufler C., Brinker A., Bourenkov G., Pegoraro S., Moroder L., Bartunik H. (2000). Structure of TPR domain-peptide complexes: critical elements in the assembly of the Hsp70-Hsp90 multichaperone machine. Cell.

[11] Haslbeck V., Eckl J.M., Kaiser C.J., Papsdorf K., Hessling M., Richter K. (2013). Chaperone-interacting TPR proteins in Caenorhabditis elegans. J. Mol. Biol..

[12] Young J.C., Barral J.M., Ulrich Hartl F. (2003). More than folding: localized functions of cytosolic chaperones. Trends Biochem. Sci..

[13] Ravalin M., Theofilas P., Basu K., Opoku-Nsiah K.A., Assimon V.A., Medina-Cleghorn D. (2019). Specificity for latent C termini links the E3 ubiquitin ligase CHIP to caspases. Nat. Chem. Biol..

[14] Dickey C.A., Yue M., Lin W.L., Dickson D.W., Dunmore J.H., Lee W.C. (2006). Deletion of the ubiquitin ligase CHIP leads to the accumulation, but not the aggregation, of both endogenous phospho- and caspase-3-cleaved tau species. J. Neurosci..

[15] Saidi L.J., Polydoro M., Kay K.R., Sanchez L., Mandelkow E.M., Hyman B.T. (2015). Carboxy terminus heat shock protein 70 interacting protein reduces tau-associated degenerative changes. J. Alzheimers Dis..

[16] Dolan P.J., Johnson G.V. (2010). A caspase cleaved form of tau is preferentially degraded through the autophagy pathway. J. Biol. Chem..

[17] Das A.K., Cohen P.W., Barford D. (1998). The structure of the tetratricopeptide repeats of protein phosphatase 5: implications for TPR-mediated protein-protein interactions. EMBO J..

[18] O'Donoghue A.J., Eroy-Reveles A.A., Knudsen G.M., Ingram J., Zhou M., Statnekov J.B. (2012). Global identification of peptidase specificity by multiplex substrate profiling. Nat. Methods.

[19] Johnson O.T., Nadel C.M., Carroll E.C., Arhar T., Gestwicki J.E. (2022). Two distinct classes of cochaperones compete for the EEVD motif in heat shock protein 70 to tune its chaperone activities. J. Biol. Chem..

[20] Simeonov A. (2013). Recent developments in the use of differential scanning fluorometry in protein and small molecule discovery and characterization. Expert Opin. Drug Discov..

[21] Wu T., Yu J.C., Suresh A., Gale-Day Z.J., Alteen M.G., Woo A.S. (2023). Conformationally responsive dyes enable protein-adaptive differential scanning fluorimetry. bioRxiv.

[22] Wu T., Hornsby M., Zhu L., Yu J.C., Shokat K.M., Gestwicki J.E. (2023). Protocol for performing and optimizing differential scanning fluorimetry experiments. STAR Protoc..

[23] Chen M.X., Cohen P.T. (1997). Activation of protein phosphatase 5 by limited proteolysis or the binding of polyunsaturated fatty acids to the TPR domain. FEBS Lett..

[24] Haslbeck V., Eckl J.M., Drazic A., Rutz D.A., Lorenz O.R., Zimmermann K. (2015). The activity of protein phosphatase 5 towards native clients is modulated by the middle- and C-terminal domains of Hsp90. Sci. Rep..

[25] Van Bibber N.W., Haerle C., Khalife R., Xue B., Uversky V.N. (2020). Intrinsic disorder in tetratricopeptide repeat proteins. Int. J. Mol. Sci..

[26] Haslbeck V., Drazic A., Eckl J.M., Alte F., Helmuth M., Popowicz G. (2015). Selective activators of protein phosphatase 5 target the auto-inhibitory mechanism. Biosci. Rep..

[27] Zeke T., Morrice N., Vazquez-Martin C., Cohen P.T. (2005). Human protein phosphatase 5 dissociates from heat-shock proteins and is proteolytically activated in response to arachidonic acid and the microtubule-depolymerizing drug nocodazole. Biochem. J..

[28] Yadav L., Tamene F., Goos H., van Drogen A., Katainen R., Aebersold R. (2017). Systematic analysis of human protein phosphatase interactions and dynamics. Cell Syst..

[29] Svejstrup J.Q. (2007). Elongator complex: how many roles does it play?. Curr. Opin. Cell Biol..

[30] Abdel-Fattah W., Jablonowski D., Di Santo R., Thuring K.L., Scheidt V., Hammermeister A. (2015). Phosphorylation of Elp1 by Hrr25 is required for elongator-dependent tRNA modification in yeast. PLoS Genet..

[31] Rapino F., Delaunay S., Rambow F., Zhou Z., Tharun L., De Tullio P. (2018). Codon-specific translation reprogramming promotes resistance to targeted therapy. Nature.

[32] Cortajarena A.L., Regan L. (2006). Ligand binding by TPR domains. Protein Sci..

[33] Oberoi J., Dunn D.M., Woodford M.R., Mariotti L., Schulman J., Bourboulia D. (2016). Structural and functional basis of protein phosphatase 5 substrate specificity. Proc. Natl. Acad. Sci. U. S. A..

[34] Jaime-Garza M., Nowotny C.A., Coutandin D., Wang F., Tabios M., Agard D.A. (2023). Hsp90 provides a platform for kinase dephosphorylation by PP5. Nat. Commun..

[35] Partch C.L., Shields K.F., Thompson C.L., Selby C.P., Sancar A. (2006). Posttranslational regulation of the mammalian circadian clock by cryptochrome and protein phosphatase 5. Proc. Natl. Acad. Sci. U. S. A..

[36] Cliff M.J., Harris R., Barford D., Ladbury J.E., Williams M.A. (2006). Conformational diversity in the TPR domain-mediated interaction of protein phosphatase 5 with Hsp90. Structure.

[37] Kampinga H.H., Andreasson C., Barducci A., Cheetham M.E., Cyr D., Emanuelsson C. (2019). Function, evolution, and structure of J-domain proteins. Cell Stress Chaperones.

[38] Yu H.Y., Ziegelhoffer T., Osipiuk J., Ciesielski S.J., Baranowski M., Zhou M. (2015). Roles of intramolecular and intermolecular interactions in functional regulation of the Hsp70 J-protein co-chaperone Sis1. J. Mol. Biol..

[39] Faust O., Abayev-Avraham M., Wentink A.S., Maurer M., Nillegoda N.B., London N. (2020). HSP40 proteins use class-specific regulation to drive HSP70 functional diversity. Nature.

[40] Li J., Wu Y., Qian X., Sha B. (2006). Crystal structure of yeast Sis1 peptide-binding fragment and Hsp70 Ssa1 C-terminal complex. Biochem. J..

[41] Gestwicki J.E., Shao H. (2019). Inhibitors and chemical probes for molecular chaperone networks. J. Biol. Chem..

[42] McAvoy T., Nairn A.C. (2010). Serine/threonine protein phosphatase assays. Curr. Protoc. Mol. Biol..

