# Supplementary material for: Exploration of the binding determinants of protein phosphatase 5 (PP5) reveals a chaperone-independent activation mechanism
Source: J Biol Chem. 2024 Jun 1;300(7):107435. doi: 10.1016/j.jbc.2024.107435 (PMC11259706; doi:10.1016/j.jbc.2024.107435)
Supplement: Supplementary information [file mmc1.pdf]

Supplementary Information

Exploration of the Binding Determinants of Protein Phosphatase 5 (PP5) Reveals a Chaperone-Independent Activation Mechanism

Shweta Devi[1], Annemarie Charvat[1], Zoe Millbern[2], Nelson Vinueza[2] and Jason E. Gestwicki[1,*]

[1]Department of Pharmaceutical Chemistry and the Institute for Neurodegenerative Diseases, University of California San Francisco, San Francisco, CA 94158 USA

[2]Department of Textile Engineering, North Carolina State University, Raleigh, NC 27695 USA

Contents:

Figure S1. Effects of optimized pentapeptides on the turnover of PP5 substrate.

Figure S2. Raw western blots for co-immunoprecipitations of ELP1 and PP5 from HEK293T cells.

Figure S3. C-terminal sequences of ELP1 orthologs across kingdoms.

A: K_m of PP5 in presence of canonical and optimized peptides

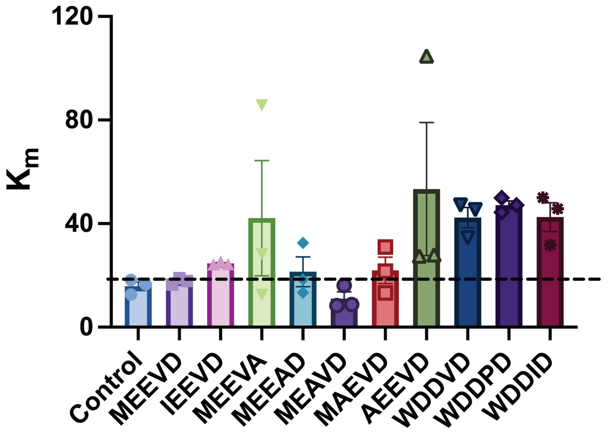

B: K_{cat}/K_m of PP5 in presence of canonical and optimized peptides

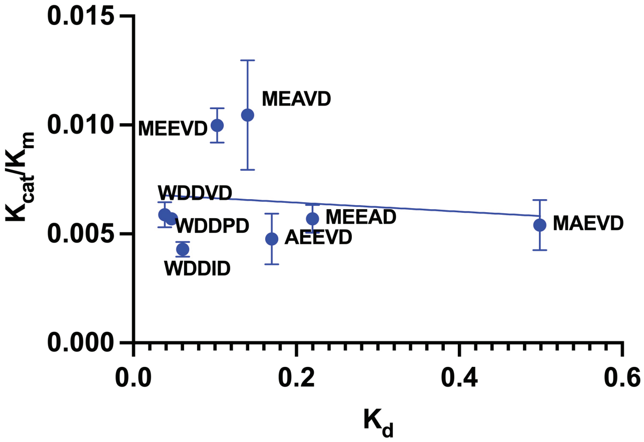

Figure S1. Effects of C-terminal peptides in the PP5 enzymatic assay. (A) Effects of peptides on the Km values. (B) Correlation between kcat/Km and the apparent affinity of peptides for PP5. All values are the average of experiments performed in two independent replicates with three technical replicates each (n = 6). The error bars represent SD.

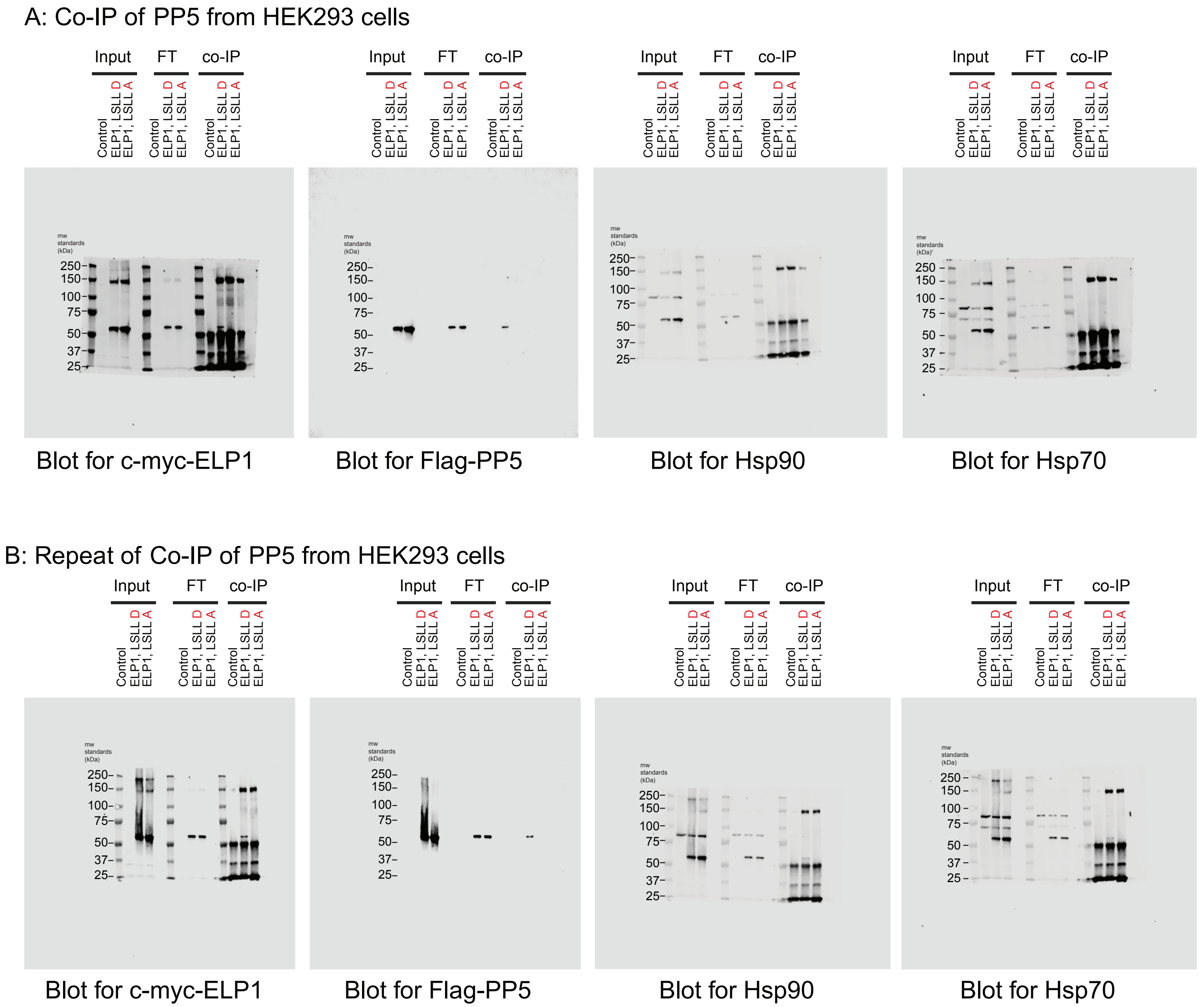

Figure S2. Raw western blots from co-immunoprecipitations of PP5 from HEK293 cells. (A) Raw, uncropped blot from the main text. (B) The raw, uncropped blot of the independent replicate. See the text for details.

Figure S3. C-terminal sequences of ELP1 orthologs across kingdoms

Fungi, Protista and Invertebrates

>Strongylocentrotus purpuratus:
LLESR
>Saccharomyces cerevisiae:
HIVDF
>Neurospora crassa:
LLGGR
>Encephalitozoon cuniculi:
LSKWL
>Cryptococcus neoformans:
FCSSM
>Arabidopsis thaliana:
VFISP
>Chlamydomonas reinhardtii:
DWGPA
>Oryza sativa:
EHAEC
>Dictyostelium discoideum:
NLTIF
>Entamoeba histolytica:
ALSLD
>C.Elegans:
ASVFP
>Ciona intestinalis:
HIYGK
>Drosophila melanogaster:
HEILQ

Verebrates

>Oryctolagus cuniculus (Rabbit):
LSLLE
>Rattus norvegicus:
LSLLE
>Mus musculus (Mouse):
LSLLE
>Sciurus vulgaris (Eurasian red squirrel):
LSMLE
>Sus scrofa (Pig):
SLLEC
>aviaporcellusGuineapig
LSLLE
>SpermophilusdauricusDauriangroundsquirr
PSHLD
>Aotus nancymaae (Ma's night monkey):
LSLLD
>Homo sapiens:
LSLLD
